# Wild pollinators enhance oilseed rape yield in small-holder farming systems in China

**DOI:** 10.1186/s12898-017-0116-1

**Published:** 2017-02-21

**Authors:** Yi Zou, Haijun Xiao, Felix J. J. A. Bianchi, Frank Jauker, Shudong Luo, Wopke van der Werf

**Affiliations:** 1 Centre for Crop Systems Analysis, Wageningen University, P.O. Box 430, 6700 AK Wageningen, The Netherlands; 2 Department of Environmental Science, Xi’an Jiaotong-Liverpool University, Suzhou, 215123 China; 3 Institute of Entomology, Jiangxi Agricultural University, Nanchang, 330045 China; 4 Farming Systems Ecology, Wageningen University, P.O. Box 430, 6700 AK Wageningen, The Netherlands; 5 Department of Animal Ecology, Justus Liebig University, Heinrich-Buff-Ring 26-32, 35932 Giessen, Germany; 6 Institute of Apicultural Research, Chinese Academy of Agricultural Sciences, Beijing, 100093 China

**Keywords:** Ecosystem services, Canola, Compensation, Honey bee, Pollination, Pollinator diversity, Wild bee

## Abstract

**Background:**

Insect pollinators play an important role in crop pollination, but the relative contribution of wild pollinators and honey bees to pollination is currently under debate. There is virtually no information available on the strength of pollination services and the identity of pollination service providers from Asian smallholder farming systems, where fields are small, and variation among fields is high. We established 18 winter oilseed rape (*Brassica napus* L.) fields along a large geographical gradient in Jiangxi province in China. In each field, oilseed rape plants were grown in closed cages that excluded pollinators and open cages that allowed pollinator access. The pollinator community was sampled by pan traps for the entire oilseed rape blooming period.

**Results:**

Oilseed rape plants from which insect pollinators were excluded had on average 38% lower seed set, 17% lower fruit set and 12% lower yield per plant, but the seeds were 17% heavier, and the caged plants had 28% more flowers and 18% higher aboveground vegetative biomass than plants with pollinator access. Oilseed rape plants thus compensate for pollination deficit by producing heavier seeds and more flowers. Regression analysis indicated that local abundance and diversity of wild pollinators were positively associated with seed set and yield/straw ratio, while honey bee abundance was not related to yield parameters.

**Conclusions:**

Wild pollinator abundance and diversity contribute to oilseed rape yield by enhancing plant resource allocation to seeds rather than to above-ground biomass. This study highlights the importance of the conservation of wild pollinators to support oilseed rape production in small-holder farming systems in China.

**Electronic supplementary material:**

The online version of this article (doi:10.1186/s12898-017-0116-1) contains supplementary material, which is available to authorized users.

## Background

A wide range of agricultural crops depend on pollination by insects [1]. The decline of pollinators in terms of abundance and species richness has caused great concern about the risk of a deterioration of crop pollination and the associated crop production [2–6]. Potential drivers for the loss of wild pollinators include habitat loss and fragmentation, insecticides, pathogens, invasive species, climate change and the interactions between them [5]. The consequences of the decline of wild pollinators for pollination services may partially be offset by managed honey bees, compensating for the loss of wild pollinators [3, 7, 8]. This view, however, has recently been challenged after assessing the contribution of wild bees, hoverflies, butterflies, moths, wasps and beetles [9, 10].

The vast majority of the studies focussing on the interplay between wild and managed pollinators in providing agricultural pollination services originates from Europe and North America, where industrialization of agriculture has resulted in agroecosystems dominated by monocultures in large fields. In contrast, Chinese agroecosystems, particular in South China, are characterised by relatively small fields, leading to a high heterogeneity in terms of crop species, field management and field edges [11]. This high heterogeneity may favour wild pollinators by providing nesting sites and floral resources [12, 13]. We therefore expect that the small-holder agroecosystems in China support a high abundance and diversity of wild pollinators contributing to pollination services that significantly exceed the contribution of managed pollinators.

A globally important crop benefitting from pollination services is oilseed rape (*Brassica napus* L.), of which China is one of the world’s largest producers with more than 7.5 million ha cultivated area for the production of cooking oil, feed and biofuel [14]. Although oilseed rape is considered a self-pollinating plant species [15], insect pollination can further increase yield and quality [3, 7, 8, 16]. Seed yield of individual oilseed rape plants is determined by the number of seeds per pod (seed set), the number of pods per plant (fruit set), and the individual seed weight. Seed set is mainly determined by the amount of pollen grains deposited on the stigma of flowers during the receptive period [17], which can be increased by pollinator-mediated pollen transfer [18]. Similarly, pollination usually enhances fruit set, i.e. the proportion of flowers developing into pods [19, 20]. Oilseed rape plants show variation in their ability to compensate for a pollination deficit, which may depend on the cultivar [21–23] and the pollination efficiency of flower visiting insects [8, 24–26]. However, the potential to compensate for pollination deficit by allocating resources into heavier seeds or increased flowering has received little attention [but see 23, 27], but may have important consequences for the oilseed rape production potential in situations of pollinator declines.

The aim of this study is twofold. First, to assess the relationship between pollinator communities and oilseed rape yield parameters. We hypothesise that yield parameters will be positively influenced by more abundant and more diverse pollinator communities. Second, to assess the relative contribution of wild pollinators versus honey bees to oilseed rape pollination. Here, we expect that yield of oilseed rape is positively associated with the abundance of both wild pollinators and honey bees.

## Methods

### Study area

We selected 18 oilseed rape fields across a large geographical area in Jiangxi Province, China (N28.35º–N28.99º, E115.26º–E115.82º). The mean distance between fields was 36.9 km (range: 5.8–75.2 km). As the maximum foraging range of most pollinator species is less than 2 km [28, 29], individual pollinators are unlikely to visit more than one field, and hence the pollinator communities in the study fields can be considered independent. The mean size of study fields was 845 ± 86 m^2^ (range: 400–1400 m^2^) and all fields were sown between the middle and the end of October 2014 with the same traditional open-pollinated winter oilseed rape cultivar YangGuang-2009.

### Experimental design and plant yield parameters

In the centre of each field, eight oilseed rape plants at a similar growth stage were selected, spaced 4 m apart. Each plant was covered by an individual cage (alternating open and closed). Closed cages had a base of 0.6 × 0.6 m^2^, a height of 2.0 m and were entirely covered with 1 × 1 mm^2^ mesh to exclude pollinators. This mesh size has only a limited influence on the microclimate in the cage [30]. The open cage was set as a control treatment and consisted of a similar frame as the closed cage, but only contained mesh at the roof and the top 0.3 m such that pollinators had access to the plants. This resulted in a similar shading of plants in closed and open cages. Neighbouring plants were removed to provide space for setting up cages, and the cages were established about one week before blooming and were removed during harvest.

After harvest, the number of pods and total number of flower stalks were counted for each plant. Seeds were removed from pods, weighed and counted using an automatic seed counter (SLY-C, Zhejiang Top Instrument, China). The following yield parameters were measured and calculated per plant: seed set (number of seeds per pod), number of pods (siliques) per plant, number of flowers per plant, fruit set (pod/flower ratio), seed weight (total seed weight divided by the total number of seeds) and yield (total weight of all seeds). Plants were dried for 30 days in the greenhouse and the total aboveground dry vegetative biomass excluding seeds and pods (referred to as straw) was assessed.

### Insect sampling

The pollinator community in each experimental oil seed rape field was sampled by pan traps, which is a suitable method for sampling pollinators such as bees [31], hoverflies [32] and butterflies [33]. Each pan trap station consisted of three cups (8.3 cm diameter, 13.5 cm height and a volume of 450 ml) fixed on a wooden stick at a distance of 1.5 m above the ground. The cups were white from the outside, and painted ultraviolet (UV) yellow, UV blue and UV white from the inside, respectively [34]. We used water saturated with kitchen salt (NaCl) as a killing agent with several drops of detergent to break water surface tension. At 3 cm from the brim, two 3 mm diameter holes were drilled in order to drain off rainwater and sufficient water was added to prevent the traps from drying out. In each field, four stations were installed at the corners of a 20 m × 20 m square in the centre of the field. Traps were set up before the onset of bloom, at the same time as the cages, and were monitored until harvest for a period ranging from 49 to 52 days. This difference in the sampling period was mainly caused by different trap establishment dates, but since the traps were established before the activity period of most pollinators, there was a negligible effect on the catch. Samples were collected five times at approximately 10-day intervals.

Insect pollinator specimens were collected and stored at −20 °C, and then sorted and pinned. All specimens were identified to species level when possible. Pollinator specimens were separated into wild pollinators (including wild bees, hoverflies, butterflies and moths, social and solitary wasps) and honey bees (*Apis mellifera* and *Apis cerana*). Asynchronous flowering times of the oilseed rape crops prevented the separation of insect communities that were visiting the oilseed fields during and after flowering (see flower cover data in Additional file 1). Therefore, all specimens collected from the same field were pooled in the analysis.

### Data analysis

We conducted two main analyses. The first analysis focused on the effect of pollinator exclusion on plant yield parameters using linear mixed effect models. Response variables were calculated as the difference in plant yield parameters between plants with pollinator access (open cages) and plants without pollinator access (closed cages), and included (1) seed set, (2) number of pods per plant, (3) number of flowers per plant, (4) fruit set (pod/flower ratio), (5) thousand seed weight, (6) plant yield, (7) straw biomass, and (8) yield/straw ratio. Plant yield/straw ratio is the ratio between total seed weight and total dry vegetative biomass. It expresses the ratio of assimilates to seeds or vegetative growth, and provides a useful indicator for limitation in active seed sinks on the plant [35, 36] as a result of pollination deficit of oilseed rape. Treatment (closed cage versus open cage) was used as an explanatory variable (fixed factor) and study field as a random factor. Transformations were applied for response variables to meet normal distribution requirements.

The second analysis focused on the effect of pollinator abundance and diversity on yield parameters. Generalized linear mixed effect models were used with study field as a random factor. Data from the open and closed cage treatments were analysed separately. The purpose of the analysis on open cages was to assess the role of different pollinator taxa in pollination, while the analysis on closed cages was conducted to verify that pollinator abundance and community composition did not affect plant yield parameters in closed cages. Response variables included (1) seed set (Gaussian error distribution with identity-link function), (2) fruit set (gamma error distribution with log-link function), (3) thousand seed weight (gamma error distribution with log-link function), and (4) yield/straw ratio (gamma error distribution with log-link function). Explanatory variables included (a) wild pollinator abundance, (b) honey bee abundance, (c) wild pollinator diversity, and (d) plant straw biomass. Study field was included as a random factor. Wild pollinator diversity was characterised in terms of the back-transformed Shannon entropy index [37], the rarefied number of species (n = 54) [38] and the Fisher’s alpha index [39]. As the back-transformed Shannon entropy index was strongly correlated with both the rarefied number of species (Pearson r = 0.94, *P* < 0.001) and Fisher’s alpha (r = 0.91, *P* < 0.001), and is a robust indicator for mobile insects and uneven sample sizes [40–42], we selected it as an indicator for pollinator diversity (with a focus on species richness) in the statistical analysis. Plant straw biomass was included as a control variable to account for variation in plant size, but was excluded for the analysis of yield/straw ratio.

All models were validated by checking residuals according to the protocol of Zuur et al. [43] and deviance residuals met normality and homoscedasticity assumptions. In addition, model residuals were checked for spatial autocorrelation using Moran’s I coefficient [44]. No significant spatial autocorrelation was found in the fitted models (*P* > 0.05). All calculations and analyses were conducted in R (v3.1.2) [45] using the “nlme” package for linear mixed effect models [46], the “lme4” package for generalized linear mixed effect models [47], and the “ape” package for spatial autocorrelation [48]. Means and standard errors are reported throughout the paper.

## Results

### Pollinator community

A total of 5148 specimens representing 60 pollinator species were collected from the pan traps. These included 3931 Hymenoptera comprising 44 species, 52 hoverflies (7 species), and 1165 Lepidoptera (9 species). The top five most abundant species were cabbage butterfly (*Pieris rapae*), two wild bee species *Eucera chinensis* and *Lasioglossum proximatum,* and two honey bee species *A. mellifera* and *A. cerana*, accounting for 21.6, 20.8, 16.5, 9.1 and 8.1% of the overall specimens, respectively (see complete species list in Additional file 1). The overall abundance of collected wild pollinators across the 18 fields was 237 (±40) individuals, ranging from 54 to 720 individuals per field, while the abundance of honey bees averaged 49 (±12) individuals, ranging from 1 to 195 individuals per field, highlighting substantial between-field variation. This variation allows a meaningful analysis of the relationship between pollinator abundance and diversity on the one hand and plant yield parameters on the other.

### Plant yield parameters

**Fig. 1:**
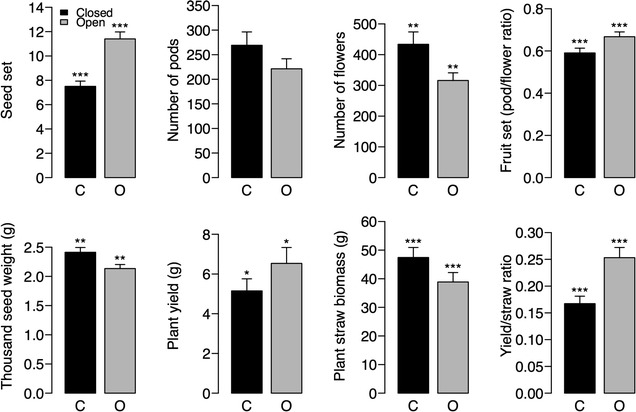
Plant yield parameters of oilseed rape plants in closed (*C*) and open (*O*) cages. *Bars* represent SEM. *Asterisks* show the significance level based on an analysis with mixed models (see Table 2; *≤0.05; **≤0.01; ***<0.001)

Results indicated that pollinator exclusion significantly influenced plant yield parameters (Table 1). Oilseed rape plants in closed cages had 38% (±4%) lower seed set, 17% (±4%) lower fruit set, 12% (±14%) lower yield and 35% (±7%) lower yield/straw ratios than plants in open cages (Fig. 1). However, plants in the closed cage treatment had 22% (±7%) higher seed weight, 28% (±9%) more flowers, and 39% (±11%) more straw biomass than plants in open cages. The number of pods per plant was not significantly different between treatments.

**Table 1:** Results of linear mixed effect models showing the effects of pollinator exclusion on oilseed rape yield parameters

Response variable	Data transformation	df	*t*	*P*
Seed set	None	113	7.86	<0.001
Number of pods	Square root	115	−1.06	0.3
Number of flowers	Square root	115	−2.99	0.003
Fruit set	Arcsine	115	4.26	<0.001
Thousand seed weight	Logarithm	111	−3.25	0.002
Plant yield	Logarithm	111	2.31	0.02
Plant straw biomass	Square root	115	−3.72	<0.001
Yield/straw ratio	Square root	111	5.21	<0.001

Negative *t* values indicate a higher value for the plants with pollinator exclusion than for the plants with pollinator access

### Influence of pollinators on plant yield parameters

When pollinators had access to oilseed rape plants, seed set was positively associated with wild pollinator abundance and diversity (Table 2). In addition, a strong positive association was observed between the yield/straw ratio and the abundance and diversity of wild pollinators. In contrast, the abundance of honey bees was not significantly associated with any of the plant yield parameters (Table 2). The control analysis using the data from the closed cage treatment did not reveal any significant effects of the abundance or diversity of pollinators on yield components, indicating that the exclusion treatment functioned well and confirming that associations between wild pollinators and yield components of oilseed rape in the open cages can indeed be attributed to insect pollination.

**Table 2:** Results of generalized linear mixed effect models showing the relationship between plant yield parameters and pollinator variables for oilseed rape plants with pollinator access (open cage)

Response variable	Error distribution	Straw biomass	Wild pollinator abundance	Wild pollinator diversity	Honey bee abundance
Seed set	Gaussian	–	0.015 ± 0.007*	0.801 ± 0.352*	–
Fruit set	Gamma	–	–	–	–
Seed weight	Gamma	–	–	–	–
Yield/straw ratio	Gamma	/	0.003 ± 0.001***	0.159 ± 0.053**	–

Values indicate estimates and standard errors, a dash (*–*) indicates that the variable was not significant, a slash (/) indicates that the variable was not entered in the model because of dependency on the response variable, and asterisks show significance levels (*≤0.05; **≤0.01; ***<0.001)

## Discussion

The heterogeneous landscape mosaic in Southern China, which is characterized by small field sizes, harboured a rich pollinator community. Accordingly, wild pollinators contributed substantially to oilseed rape yield, confirming our expectations. The fact that we did not find statistical support for the contribution of honey bees to oilseed rape yield parameters, despite their well-documented contribution crop pollination in other parts of the world, substantiates the relevance of natural service providers to small-holder farming.

We identified 60 insect pollinator species, 44 of which were Hymenoptera species. This represents a high number of pollinator species in comparison with other landscape-scale studies in oilseed rape. For example, 20 species (honeybees, bumblebees and solitary bees) from 1181 individuals were reported in Wiltshire, UK [49], 36 flower-visiting species from 1866 individuals Uppsala, Sweden [3], and 26 bee and hoverfly species in Ireland (number of individuals was not mentioned) [8]. Our findings were in line with our expectation that the study region contains a high diversity of wild pollinators, which may partly due to the high heterogeneity in field size, crop species and crop management, and high diversity of wild plant species in field edges in small-holder agroecosystems [11].

Insect pollinated plants showed higher seed set and overall higher yields than plants deprived from pollinators. Therefore, our study contributes to a body of evidence that insect pollination matters for oilseed production despite its capacity for self-pollination [3, 21, 50–52]. At the same time, insect pollination lowered some yield parameters such as seed weight, suggesting compensation mechanisms of the plants also in line with previous studies [19, 21]; but see Bommarco et al. [53]. Often, plants with a pollination deficit produce fewer seeds per pod, but each seed then receives a higher share of the plant assimilates [19, 21, 52, 53]. The higher number of flowers on plants in closed as compared to open cages provides further support for compensatory responses to a pollination deficit [23].

The higher straw biomass of oilseed rape plants in closed cages points to an increased allocation of assimilates to the above ground vegetative plant parts, which supports a lack of sink strength resulting from a pollination deficit. The positive effect of pollinator exclusion on straw weight may also in part be due to the high energetic cost of producing fatty acids in seeds as compared to the lower energetic conversion costs to leaf and stem dry matter [54]. Overall, compensation effects did not fully counterbalance the yield loss due to the lack of pollination as exemplified by the 12% higher yield when comparing plants with pollinator access to plants without pollinator access.

Wild pollinator abundance and diversity were positively associated with oilseed rape seed set and yield/straw ratio, but not with fruit set and seed weight, suggesting that their benefits to oilseed rape yield per plant mostly result from an increased number of seeds per pod. The control analysis, which showed no relationship between plant yield parameters and pollinator abundance and diversity for closed cages, confirmed the effectiveness of the exclusion treatment and the overall consistency of the experimental setup. This also suggested that pollinator collections from pan traps can be used as a proxy in reflecting the pollinator communities and pollination service at the landscape scale [55, 56].

We assessed the contribution of insect pollination on isolated plants where neighbouring plants were removed. The focus on isolated plants may have also resulted in an reduction of plant-to-plant pollen transfer [35] and a reduction in plant competition for water, nutrients and light. Therefore, we may underestimate the potential of closed oil seed rape stands to compensate for pollinator limitation by mechanical and wind pollination [35, 57] and refrain from estimating agronomic benefits at the field level [22]. Furthermore, the mesh tents may have reduced wind pollination, even though the same mesh size has been widely applied in pollinator exclusion studies [3, 16, 19, 53].

Surprisingly, our analysis gave no support for the contribution of honey bees to oilseed rape yield, even though there were large differences in honey bee abundance between fields. While the contribution of honey bees to crop pollination is widely documented [see review in 58], this result is in line with a current global meta-analysis that highlighted the importance of wild pollinators in crop pollination [9]. In our study, the higher contribution of wild pollinators to crop pollination can in part be attributed to their five times higher abundance as compared to honey bees. Indeed, wild pollinators dominate pollinator communities in many agroecosystems [59]. The relative low number of honey bees may have resulted from a relatively low density of bee hives in the study areas. Also, some wild pollinator species may be as efficient or even more efficient than honey bees [24, 26]. In our case, the abundant cabbage butterfly (*Pieris rapae*) might be an important pollinator [60]. As their larvae are considered a pest, however, this species may have both a positive and negative effect on oilseed rape production.

## Conclusion

Our study demonstrates that wild pollinators play an important role in the pollination of oilseed crops in small-holder farming systems in China. Wild pollinator abundance and diversity contribute to oilseed rape yield by mediating increased allocation to seeds rather than above-ground straw biomass, but oilseed rape plants suffering from a pollination deficit can compensate to some extent by generating heavier seeds, more flowers and higher straw biomass. This study highlights the importance of conserving wild pollinators in order to maximise oilseed rape production, especially in heterogeneous landscapes where their pollination service is exceeding the service provided by managed pollinators.

## Additional file

**Additional file 1.** Raw data on plant yield components, pollinator abundance and pollinator names.
